# Policy context, coherence and disjuncture in the implementation of the Ideal Clinic Realisation and Maintenance programme in the Gauteng and Mpumalanga provinces of South Africa

**DOI:** 10.1186/s12961-020-00567-z

**Published:** 2020-06-03

**Authors:** Immaculate Sabelile Muthathi, Laetitia C. Rispel

**Affiliations:** 1 School of Public Health, Faculty of Health Sciences, University of the Witwatersrand, 27 St Andrews Road, Parktown, Johannesburg, 2193 South Africa; 2 Centre for Health Policy & Department of Science and Innovation/National Research Foundation Research Chair, University of the Witwatersrand, 27 St Andrews Road, Johannesburg, 2193 South Africa

**Keywords:** Policy implementation, Bressers’ theory, context, intergovernmental relations, ideal clinics, South Africa

## Abstract

**Background:**

Universal health coverage is a key target of the Sustainable Development Goals and quality of care is fundamental to its attainment. In South Africa, the National Health Insurance (NHI) system is a major health financing reform towards universal health coverage. The Ideal Clinic Realisation and Maintenance (ICRM) programme aims to improve the quality of care at primary healthcare level in preparation for NHI system implementation. This study draws on Bressers’ Contextual Interaction Theory to explore the wider, structural and specific policy context of the ICRM programme and the influence of this context on policy actors’ motivation, cognition and perceived power.

**Methods:**

This was a nested qualitative study, conducted in two NHI pilot districts in the Gauteng and Mpumalanga Provinces of South Africa. Following informed consent, we conducted in-depth interviews with key informants involved in the conceptualisation and implementation of the ICRM programme. The questions focused on ICRM policy context, rationale and philosophy, intergovernmental relationships, perceptions of roles and responsibilities in implementation, ICRM programme resourcing, and implementation progress, challenges and constraints. We used thematic analysis, informed by Bressers’ theory, to analyse the data.

**Results:**

A total of 36 interviews were conducted with key informants from national, provincial and local government. The wider context of the ICRM programme implementation was the drive to improve the quality of care at primary healthcare level in preparation for NHI. However, the context was characterised by contestations about the roles and responsibilities of the three government spheres and weak intergovernmental relationships. Notwithstanding examples of strong local leadership, the disjuncture between two national quality of care initiatives and resource constraints influenced policy actors’ experiences and perceptions of the ICRM programme. They expressed frustrations about the lack of or diffuse accountability and their lack of involvement in decision-making, thus questioning the sustainability of the ICRM programme.

**Conclusions:**

National health sector reforms should consider the context of policy implementation and potential impact on actors’ motivation, cognition and power. All relevant policy actors should be involved in policy design and implementation. A clear communication strategy and ongoing monitoring and evaluation are prerequisites for implementation success.

## Background

Universal health coverage (UHC) is a key target of the Sustainable Development Goals [1, 2]. During 2018, three global health reports recommended that quality improvements are essential for the realisation of the benefits of UHC [3–5]. All reports underscored the vulnerability of people in low- and middle-income countries (LMICs) to poor quality care. The authors called on governments in LMICs to prioritise health system-wide quality of care improvements [3–5].

South Africa is a constitutional democracy and health services are a concurrent function of both national and provincial governments [6]. The national government is responsible for policy development, resource allocation, and monitoring and evaluation, whereas the provincial government is the main implementing agency [7]. The local government is responsible for municipal health services, defined in the National Health Act as environmental health services [7]. These constitutional responsibilities influence both policy context and implementation. The National Health Insurance (NHI) system is a major health financing reform towards UHC, while concomitantly addressing the inequities in the two-tiered healthcare system [8]. The latter consists of a resource-constrained public health sector that provides care to approximately 83% of the South African population and a private health sector that caters for a minority (~17%) of the population with private health insurance [8]. The NHI also aims to address quality, access, rising healthcare costs, efficiency and effectiveness through a series of complementary health sector reforms [9]. One of these reforms was the establishment of the Office of Health Standards Compliance (OHSC) in 2014, through an amendment of the National Health Act [9]. The OHSC is a legal entity that aims to protect and promote the safety of users of health services by ensuring that health establishments comply with the national quality core standards (NCS) and that complaints about healthcare are investigated and action is taken where necessary [9].

The National Department of Health (NDoH) prioritised reforms at the primary healthcare (PHC) level as part of the first phase of the NHI system implementation. The NDoH commenced an initiative called the Ideal Clinic Realisation and Maintenance (ICRM) programme. The stated goal of the ICRM programme is to prepare all PHC facilities to meet the quality standards set by the OHSC [10]. An ideal clinic is defined as a clinic with “*good infrastructure, adequate staff, adequate medicines and supplies, good administrative processes, with sufficient bulk supplies and it uses applicable clinical policies, protocols and guidelines, and it harnesses partner and stakeholder support*” ([11], p. 11). The implementation of the ICRM programme entails using the ICRM tools with indicators for quality to assess and grade facilities on a quarterly basis. Following each assessment, quality improvement plans are generated and provided to the PHC facility managers to guide their improvement actions [12]. The process is cyclical, beginning with self-assessments by PHC facility managers, followed by district assessments and, finally, the inter-district assessments [12]. The desired outcome is the achievement of ‘ideal clinic status’, which implies that the PHC facility has met the required quality standards [10].

Hence, both the OHSC, which has legal enforcement powers, and the ICRM programme share a common goal of improving the quality of care provided and the experiences of health service users. However, both the ICRM programme and the NCS of the OHSC are implemented in the same PHC facilities. We could not find any empirical studies on the coherence, synergies and/or differences between the two quality improvement initiatives.

This study draws on policy implementation theory to explore the policy context of the ICRM programme, given the existence of the OHSC as a legal entity. We refer to policy context as the background against which policy decisions are made, policy processes take place and stakeholders or actors engage with the policy [13]. Policy coherence is the the ability of similar policies to reinforce one another, create synergies towards one goal (in this case quality improvement) while providing actors with clear guidelines to address the policy problem [14]. Policy implementation theories are suitable for use in this study because the implementation of the ICRM programme is a major PHC policy reform [15].

The ICRM programme commenced in 2013 and there is a dearth of empirical studies on implementation. Although the NDoH reported that the ICRM programme has had positive impact in improving waiting times and stock availability [16], progress is uneven, with a reported 56% of clinics meeting the ideal clinic criteria [17]. Importantly, the NDoH has expressed concerns that approximately 25% of all the PHC clinics that were ICRM programme compliant, had lost their ideal clinic status in a 4-year period [18].

Policy implementation studies are important, especially in LMICs, because a lack of, or poor, implementation could result in financial waste, a lack of service delivery, frustration and loss of confidence in government [19–21]. Research on policy implementation also plays an important role in highlighting the progress and challenges of government policies, thereby enhancing public accountability [22]. The analysis of policy context explains the reasons for putting an item on the policy agenda [23], namely the feasibility, relevance and appropriateness of the policy [24]. Such analysis also provides insights into the potential implementation challenges [25] and the likely sustainability of the policy initiative [26].

Various researchers have examined the interplay between context and the role and characteristics of policy actors [27–29]. In Colombia, the implementation of HIV and AIDS policies were undermined by the lack of political will, the power of politicians at local government and corruption in resource distribution [30]. In Malawi, a study on the implementation of environmental health policy in healthcare facilities was adversely affected by poor co-ordination and lack of information [31]. A study on the implementation of healthcare financing policies in South Africa and Zambia found that inadequate communication and lack of consultation with various policy actors resulted in unanticipated and unwanted impacts [28].

In South Africa, studies on the implementation of the water sector skills plan [32] and free basic energy policy [25] found that a lack of consultation with stakeholders, insufficient information, capacity gaps and demotivated front-line staff contributed to the lack of prioritisation and slow progress during implementation. Other studies in the health sector examined the implementation of public sector financial incentives [33, 34], nursing education reforms [35, 36] and nurses’ participation in policy-making [37]. These studies found that careful planning, coordination, proactive leadership, resources and skills facilitated policy implementation [33, 34]. In contrast, insufficient understanding of policy goals, poor communication, resource constraints and poor or non-existent monitoring and evaluation hampered implementation [36, 37].

The provision of high quality UHC in South Africa is a national priority [38], hence it is essential to ensure the successful implementation of quality of care reforms. The ICRM programme is designed to address the deficiencies in the quality of PHC services [39], and “*to lay a strong foundation for the implementation of the NHI*” ([40], p. 1). A study to analyse its implementation could provide insights on the contextual facilitators and/or constraints in order to propose recommendations on how to improve future implementation of the ICRM programme. The results could also inform or contribute lessons for the design and implementation of other NHI reforms in South Africa. Hence, the purpose of this study was four-fold. Firstly, we aimed to generate new knowledge on the policy context of the ICRM programme implementation. Secondly, we wanted to examine the influence of context on the motivation, cognition and perceived power of the policy actors and on how those interactions influenced ICRM implementation. Thirdly, we wanted to explore whether there is policy coherence in the ICRM programme and the OHSC as both these reforms focus on health system-wide quality improvements. Lastly, we wanted to explore the perceptions of stakeholders at national, provincial and local government on these two quality improvement reforms. The research is part of a larger doctoral study on the implementation of the ICRM programme in two South African provinces.

## Methods

### Conceptual framework

**Fig. 1:**
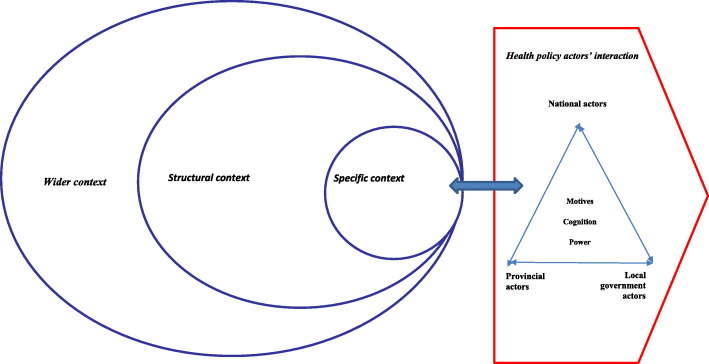
Conceptual framework adapted from Bressers’ Contextual Interaction Theory. Source: Bressers (2009); adapted with permission from authors [41]

Although there are numerous policy implementation theories and frameworks, we chose Bressers’ theory [41] to examine the policy context of the ICRM programme implementation (Fig. 1). The theory is suitable for this study because it allows us to examine both the context of policy implementation and the policy actors’ motivation, cognition and power, and how these aspects influenced the implementation of the ICRM programme.

Bressers’ theory categorises context into three layers as specific, structural and wider (Fig. 1). The specific context refers to previous policy decisions made and the influence of geographical location on the implemented policy [41]. In our study, previous policy decisions refer to the NCS of the OHSC, used initially to assess the quality standards of the PHC facilities prior to the introduction of the ICRM programme. Geographical location refers to a facility in rural or urban areas. Structural context refers to the actors’ position in governance, their interaction with other actors, actors’ roles and responsibilities, and the availability of resources [42]. In our study, structural context takes account of the three spheres of government involved in the ICRM implementation, the actors’ roles and responsibilities, their interaction, and the resource availability and challenges in implementing the ICRM programme. Wider context refers to the cultural, political and economic issues that influence the implementation [41]; in our study, the wider context refers to the conceptualisation of the ICRM programme and its subsequent implementation.

The overall context influences the actors’ characteristics of motivation, knowledge, and power and these characteristics shape and re-shape the policy implementation process [41]. Cognition refers to the interpretation of the reality by policy actors and their frames of reference [43]. In our study, it refers to the views and experiences of actors regarding the ICRM implementation. Motivation refers to both the intrinsic and extrinsic motivation of policy actors [43]. Power refers to the policy actors’ access to resources and/or possession of legal rights and their capacity to influence decisions and implementation [43].

We used Bressers’ theory [41] in the design of the data collection tools, such as in the phrasing of the questions in the interview schedule and to inform the inductive, thematic analysis of the in-depth interviews.

### Study setting

The various NHI projects, including the ICRM programme, were piloted in 11 of the 52 health districts (municipalities) of South Africa [44]. Our study setting was in two of the NHI pilot districts, the City of Tshwane district in Gauteng Province (GP) and Gert Sibande district in Mpumalanga Province (MP).

GP is an urban province, contributing to around one-third of South Africa’s gross domestic product [45]. GP is divided into five municipalities or health districts. The City of Tshwane district, which is a large metropolitan municipality, owns and manages 24 of the 74 PHC facilities [46]. The City of Tshwane metropolitan municipality has a service level agreement with the Gauteng Department of Health (GDoH) to provide health services on its behalf [47].

MP is predominantly rural province and is divided into three municipalities or health districts. All the 77 PHC facilities are managed by the provincial government [48].

The criteria for the selection of these two provinces were their designation as an NHI pilot district, geographical proximity, and budgetary and logistical considerations. In this study, the GDoH and the Mpumalanga Department of Health represented the provincial sphere of government and the City of Tshwane municipality represented the local sphere of government.

### Study design

This was a qualitative study nested in a larger mixed methods doctoral study. The latter uses policy implementation theory to analyse the implementation of the ICRM programme in GP and MP.

### Participant selection

We used purposive sampling to select key informants. Key informants had to meet one or both of the following criteria: leadership in the ICRM programme at national, provincial or local government or in the NHI pilot districts, and active involvement in the ICRM conceptualisation and/or implementation.

### Data collection instrument

We developed an interview schedule in English, which is the official business language in South Africa. The interview schedule consisted of semi-structured questions, informed by Bressers’ theory [41] and the research objectives. The semi-structured questions were categorised into six sub-sections, namely the ICRM policy context, rationale and philosophy, intergovernmental relationships, perceptions of roles and responsibilities in ICRM implementation, resourcing of the ICRM programme, implementation progress, and challenges and constraints.

A team of health systems researchers reviewed the interview schedule for content validity and the clarity of questions. The principal researcher (PR), a PhD candidate in Public Health with training and experience in qualitative research, piloted the interview schedule with two key informants from a different health district to determine the clarity of questions and the time taken to complete the interview. Following feedback from the pilot interviews, the PR re-arranged two questions to enhance the flow of the interview, prior to the actual fieldwork, but no substantive changes were necessary.

### Data collection

The PR contacted each participant via email to request voluntary participation in the study. Following informed consent, the PR arranged the interview date and time with each key informant.

The PR conducted all interviews in English. The interview began with an introduction to the study, and an explanation of the voluntary nature of participation. Following informed consent, the PR used the semi-structured interview schedule as a guide to explore each participant’s perceptions of the ICRM programme implementation. Each interview lasted for about an hour, although the duration varied depending on the responses provided by the key informant.

Interviews were recorded digitally and labelled with a key informant code. Immediately after the interviews, the PR wrote a synopsis of each interview to support data analysis. All audio-recordings are kept on a password-protected computer to ensure confidentiality and only the PR has access to the data.

### Data analysis

The interviews were transcribed verbatim. The PR cleaned the data through reading each transcript while listening to the original recording. Following data cleaning, the PR invited two additional researchers to assist with data analysis to achieve intercoder agreement, one a health systems researcher with expertise in qualitative data analysis and the other the PR’s research supervisor. Two diverse transcripts were given to the researchers involved in data analysis. We agreed to use inductive coding with direct words from participants to name codes and followed thematic analysis [49] to analyse the data. Coding was performed independently by each researcher and we held a coding meeting to discuss and agree on the codes.

Following the coding meeting, the PR tabulated all the codes from the two transcripts and analysed them for recurring patterns of meaning and contradictions within and between transcripts. Using participants’ words from the codes, the PR developed themes by writing text segments reflecting meaning of the data. The PR also interrogated and evaluated themes for similarities and differences in meaning across different levels of government and across provinces, and compared the generated themes against Bressers’ theory and vice versa.

The PR then distributed the developed themes to the other two researchers who had coded the data for review and validation of the final themes. After agreeing on the final themes, the PR analysed the rest of the data.

### Trustworthiness and rigor

We applied Lincoln and Guba’s criteria of trustworthiness in the study [49]. The participation of two other researchers in data analysis ensures reliability of the findings. Attaching excerpts of narratives in the report writing to illustrate themes ensures confirmability. The iterative process of repetitive listening to the audio-records during data cleaning allowed for prolonged engagement with the data, thereby ensuring credibility. We read the synopses of interviews and used these as a reference to validate codes during the analysis of the rest of the data and to confirm the final generated themes.

## Results

A total of 36 key informants agreed to participate in the interviews and there were only two refusals with no specified reasons. The key informants who consented to participate consisted of the following groups of health policy actors: national government (*n* = 3), provincial government head office (*n* = 5), district (*n* = 9), sub-districts (*n* = 13) and local government (*n* = 6).

Table 1 shows the alignment between Bressers’ theory [41] and the inductive themes that emerged from the study. Although these overlap, we elaborate on these below.

**Table 1:** Emerging themes on the context of the ICRM programme and interplay with the characteristics of actors

Context		Actor characteristics (Cognition/Motivation/power) as influenced by the context		
	Themes	Cognition	Motivation/demotivation	Power/capacity or lack of power
Wider context	Improving quality in preparation for NHI implementation	NDoH recognised the need to improve quality in preparation for NHI	NDoH motivated to improve quality at PHC level	NDoH used legal powers to drive the ICRM programme
Structural context	Contestations about roles and responsibilities	Conflicting views on roles and responsibilities	Frustrations about lack of reporting structure and lack of accountability	Unfulfilled responsibilities by all government spheres disempowered the implementers at facility level
	Weak intergovernmental relationships	Provincial, district and local government reported ineffective communication, lack of co-operation and top-down approach of the NDoH	Lack of ownership, insufficient buy in, leading to demotivation	–
	Local leadership enables implementation	District and local government perceived their leadership knowledge and skills as facilitating the implementation	Knowledge and experience motivated the sub-district managers to implement ICRM	Managers negotiated for additional resources
	Insufficient resourcing of ICRM	Ambivalence about ICRM programme sustainability	District managers expressed urgency and advocated for spending	District and local authority staff reported lack of authority or control over NHI grant or provincial cash flow
Specific context	Gaps in existing policy	District and local government reported insufficient communication on the NCS and lack of enforcement	NDoH motivated to design ICRM programme to assist with compliance with NCS	Insufficient communication disempowered district actors
	Insufficient policy coherence or disjuncture	District and local government staff recognised the need to align tools	District and sub-district staff experienced frustration, confusion and exhaustion, which were demotivating	Experienced lack of power to influence the alignment of tools
	Geographical variations Infrastructural variations	Ambivalence about ICRM programme	District and local government reported sense of despair that some facilities will never be ideal	Perceived lack of capacity to implement ICRM in facilities with infrastructure challenges

*ICRM* Ideal Clinic Realisation and Maintenance, *NCS* National Core Standards, *NDoH* National Department of Health, *GDoH* Gauteng Department of Health, *NHI* National Health Insurance, *PHC* primary healthcare

### Wider context

The central theme on the wider context was the imperative to improve the quality of PHC in preparation for NHI implementation. The NDoH conducted an audit of PHC in 2011 [50], which revealed poor infrastructure of PHC facilities, long waiting time, shortages of medicines and lack of cleanliness, leading to patient dissatisfaction and poor utilisation of PHC facilities. Key informants at the national level were of the opinion that the facility audit results provided the major impetus for an improvement initiative, which then became the ICRM programme.

“*In 2011, we commissioned an audit of all health facilities in the country, where a team visited more than 4000 facilities in the country. The majority of the primary healthcare facilities were in a bad state, from infrastructure, waiting time, shortages of medicines, cleanliness, patient satisfaction and infection care control. We thought we could come up with a single approach to improve* [PHC] *healthcare facilities.*” (Key Informant 2, National)

The health facility audit revealed the difficulties of PHC facilities to comply with the national core standards (NCS) of the OHSC, despite ongoing assessments of these facilities. Hence, the ICRM programme sought to address the problem of non-compliance.

“*We realised that we need to do something internally to proactively improve the quality of the health facilities. If we didn’t, the Office of Health Standards Compliance would find year after year non-compliant* [PHC] *facilities. The ICRM programme is a structured systematic approach to improve quality.*” (Key Informant 1, National)

Hence, the NDoH used their Constitutional powers [6] and the provisions of the National Health Act [7] to develop a national policy for quality improvement in the PHC facilities. This cognition, combined with the problems revealed by the PHC facility audit, served as motivation for the NDoH to initiate and drive the implementation of the ICRM programme.

Key informants from both provincial and local authorities were of the opinion that the ICRM programme was conceptualised to deal with the lack of standardisation of PHC facilities across the country. They highlighted the need for uniformity of all PHC facilities irrespective of their geographical location and to increase PHC facility utilisation in preparation for the NHI system.

“*The idea was to make all facilities in the country to be the same, because if you go to Gauteng you must get the same quality of service that you get in Mpumalanga, irrespective of rural and urban variations.*” (Key Informant 25, District level, MP)

### Structural context

There were four themes that emerged, namely contestations about roles and responsibilities, weak intergovernmental relationships, enabling local leadership, and insufficient resourcing of the ICRM programme.

#### Contestations about roles and responsibilities

There were conflicting views on the roles and responsibilities, depending on the positionality of the key informants interviewed. The sub-themes that emerged included unfulfilled responsibilities, contestations about ICRM programme funding, unclear and/or lack of enforcement of roles, and lack of a reporting structure in GP.

#### Unfulfilled responsibilities

Key informants from national government indicated that their roles were to conceptualise the ICRM programme and develop the policy documents for the implementation, communicate the idea to the other levels of government, set targets for all provinces, establish teams to oversee provinces, establish a delivery unit for the ICRM programme and provide funding. Although key informants from other government spheres shared this view, they talked about the perceived failure of the National Department of Health to produce the requisite policies on time.

“*Currently, there is not a signed policy for waiting times, just a draft. Clinical audits, a draft. So, the role from National has not been clear, I mean how long did it take them to provide us a signed copy of the patient experience of care? It took few years. We received it last year* [2017]*. The Clinic Card Audit is outstanding, the referral policy is outstanding.*” (Key informant 12, Tshwane municipality, GP)

The provincial key informants were of the opinion that the role of the provincial sphere was to develop memoranda of agreement between the Provincial Department of Health and other departments (e.g. Department of Education, etc.) and to support the districts. However, those at the district level pointed to the perceived lack of ownership of the programme by the GDoH and the lack of support. Furthermore, they were of the opinion that GDoH delayed in assigning the ICRM focal person and did not perform their roles until the district was obliged to communicate directly with the NDoH.

“*In my opinion Gauteng Province hasn’t really supported us at all with the ICRM programme, in fact they saw this* [programme] *as a monster, and it’s only now recently that the ICRM programme is being spoken at the same level as the national core standards. I really don’t see what role they* [GDoH] *played in the whole process*.” (Key informant 6, District level, GP)

Similarly, in MP, some key informants indicated that the provincial committee for the ICRM programme was unable to hold meetings.

“*The short coming of our Mpumalanga province is that they are not able to have meetings of the ICRM programme. They should be meeting on a quarterly basis*.” (Key informant 23, District level, MP)

These PHC facility managers reported that the unfulfilled roles and responsibilities by various departments and levels of government created a sense of disempowerment and influenced their ability to achieve ideal clinic status.

There were also contestations about the role of the provincial health department in funding of the ICRM programme. National level key informants thought that GP should start allocating funds for the ICRM programme.

“*I think the provinces should take ownership, and apportion resources to drive the programme*.” (Key Informant 3, National)

In contrast, provincial level key informants considered the ICRM programme as an unfunded mandate and thought that it was the NDoH’s responsibility to fund the ICRM programme.

“*It would have been ideal for provinces to fund, but that was an unfunded mandate on our side. We do the* [financial] *planning in advance and this* [ICRM] *programme was not in the budget process.*” (Key informant 3, Provincial level, GP)

All key informants agreed that the local government and district level of provincial government were responsible for the actual implementation of the ICRM programme. Such implementation included facility assessments, assisting PHC facility managers to identify deficiencies in their facility and to develop quality improvement plans to support achievement of the required standards. Furthermore, local government and districts were responsible for resource motivation and negotiation to support the PHC facilities, and to support the maintenance of ideal facility status. However, the local government in GP was criticised for lack of cooperation and at times refusing to implement provincial instructions. One key informant pointed to the failed discussions over provincial government branding of all the facilities, including those managed by the local government, and on the local government’s refusal to provide signage to PHC facilities. The latter is an important criterion in the implementation of the ICRM programme.

“*Some of the challenges are issues like branding of a clinic. The municipalities* [local government]*, insist to use their own brand. The ICRM programme requires that every province should use the provincial premier for their clinics but then you don’t have that kind of buy-in from the municipalities.*” (Key informant 5, District level, GP)

#### Role clarity

Although the majority of key informants thought that the roles were clear and well described in the ICRM programme manual, some thought there was lack of enforcement of roles and responsibilities, and unclear responsibility and lack of accountability, which affected PHC service delivery. Those who described roles as unclear pointed to overlapping roles and an unclear reporting structure.

“*They* [roles] *are clear except the enforcement because while the responsibilities are clear in terms of performance on the ICRM programme. The facility gets punished for things they're not responsible for.*” (Key informant 5, District level, GP)

In GP, some of the key informants were of the opinion that there is a lack of role clarity between national, provincial district health services and the provincial quality assurance departments.

“*You have the national; you have the* [provincial] *quality assurance unit which is not part of the ICRM programme. Then you have the* [provincial] *district health services unit which is responsible for primary health care in the province, now the question is who then takes responsibility between the two of them?*” (Key informant 6, District level, GP)

In MP, a key informant was of the opinion that there were overlapping roles and responsibilities:

“*I think the roles are overlapping and that they cause a situation of when a function is not clearly designated ….it makes everyone run around doing the same thing.*” (Key informant 24, District level, MP)

In GP, some key informants were of the opinion that there was an unclear reporting structure for the NHI district. Consequently, the pilot district liaised directly with the NDoH and GDoH was kept informed on a need to know basis.

“*In Gauteng province, we did not have an NHI coordinator. When we started, National* [NDoH] *appointed directors to assist with that process at a provincial level. There was no one appointed for Gauteng. Because the piloting was happening in Tshwane, one of Tshwane managers was taken as a coordinator of NHI in the province by virtue of being in a pilot site. There was no structure in the province to hold Tshwane district as an* [NHI] *pilot site accountable.*” (Key informant 5, District level, GP)

In GP, the lack of role clarity resulted in some policy actors at district level bypassing the provincial level and requesting resources from NDoH. In MP, the lack of role clarity reportedly caused delays in procuring equipment for the facilities and confusion on the execution of certain tasks.

“*Because when we wait for equipment from national, sometimes you submit this list in March this year until next year March you continue to wait, we continually score zero for certain equipment. We cannot buy it because we’ve sent already the list to national*”*.* (Key informant 24, District, MP)

#### Weak intergovernmental relations

Key informants pointed to relatively poor relationships across government spheres, characterised by a perceived top-down approach of the NDoH, ineffective communication and lack of cooperation, including disagreements on goals and ambitions. The perceived weak interrelationships between government levels and between departments decreased policy actors’ sense of ownership of the ICRM programme.

“*It* [ICRM programme] *was forced on us. It has been pushed down to us without really proper communication…. like all the other* [health] *programmes.*” (Key informant, Tshwane municipality, GP)

There was also the perception that the NDoH did not consider the views of people from local government.

“*This concept is actually driven from the National space. If we could have had an opportunity to say ‘let’s hear the views from the implementing partners’ meaning your locals, your districts, and so on and so forth, maybe we could have actually worked it out in a different way.*” (Key informant 7, Tshwane Municipality, GP)

The insufficient involvement of implementers in conceptualising the ICRM programme led to a lack of buy-in.

“*Initially we also didn’t have ownership and buy-in in of the ICRM programme. Because they* [clinic staff] *also felt that it’s forced down on us, expecting us to come with a prescribed checklist expecting all these things from us.*” (Key informant 11, Tshwane municipality, GP)

Furthermore, the nature of communication across the three government spheres was described as rigid, hierarchical and uncoordinated, at times not reaching sub-districts and PHC facilities.

“*Information comes from national to the province, and the province sends it to the district, and the district must send down. When you communicate from the province you should send information to the person at the district and copy those people at facilities so that they are on board, but that doesn’t happen. It leaves those at the ground level uninformed.*” (Key Informant 9, District level, MP)

“*National is communicating with Provinces, Provinces should communicate with local government and the districts. But Gauteng province doesn’t do that, they communicate with the district and the district then has to communicate with the local authority. That was a challenge.*” (Key informant 6, District level, GP)

Some key informants from the provincial level voiced their opinions about an uncooperative local government.

“*The municipality has this thing that,‘hey, I’m my own boss. We are an independent sphere of government. They* [Gauteng province] *are not going to tell us what to do’. Sometimes you find that when you’re communicating, making requests,* [or], *advising on something, the response takes forever*”*.* (Key informant 3, Provincial level, GP)

In contrast, some key informants were of the opinion that there were disagreements on goals and ambitions of national and provincial departments of health, with each pursuing its own quality of care initiative, e.g. national implementing ICRM programme and the provincial quality assurance focused on the NCS.

“*Even up to now, the ICRM programme is seen as a programme of district health services. Quality assurance in the province has not bought into the ICRM programme. They're pursuing the national core standards; they couldn't be bothered with that* [ICRM] *programme.*” (Key informant 5, District level, GP)

#### Enabling local leadership

Key informants underscored the importance of local leadership in enabling successful ICRM programme implementation. Those facility managers with extensive and wide-ranging PHC experience, management, clinical care as well as passion for quality assurance, enabled successful ICRM programme implementation.

“*I trained in primary healthcare in 1985. Then, I became a clinic manager, at that time I managed two clinics…my practical experience of working in the facilities contributed to my knowledge of what is required, and …but also my love for, always trying to do the right thing.*”(Key Informant 10, Tshwane municipality, GP)

These experienced and knowledgeable managers were both motivated and used their agency to negotiate for additional resources, or to share resources with other PHC facilities.

“*We do assessments and identify gaps, thereafter we mobilise resources within sub-districts. When one facility doesn’t have a required item and you see that another facility has two, you ask that facility to share.*” (Key Informant 31, Sub-district, MP)

“*We keep escalating the identified challenges; we make recommendations out of the gaps and influence the following year’s budget projection.*” (Key informant 12, Sub-district, GP)

#### Insufficient resourcing of the ICRM programme

Key informants indicated that the NHI conditional grant was only allocated 2 years after the commencement of the ICRM programme. Once they obtained the conditional grant, there were spending challenges because of the conditionality of the grant and insufficient consideration of the needs of the ICRM programme. In MP, the spending challenges were also exacerbated by the hospital-based supply chain, which did not prioritise PHC needs.

“*There were challenges. It* [the grant] *had so many limitations, that’s why in all provinces, the first grant was not spent. They* [NDoH] *will tell you, you couldn’t buy equipment, the fund is for training*.” (Key informant 5, District level, MP)

“*The biggest problem is, even if you can give me a 100 million rand now* [USD 8 million, 1 USD=R15]*, to say get these 10 PHC facilities to be ideal. Because it’s government money, I have to have use supply chain and in our case* [Mpumalanga province] *supply chain that is available is at the hospital.*” (Key informant 27, District level, MP)

The City of Tshwane District indicated that accessing resources was a challenge due to limited delegations, cash flow problems at the Gauteng province and the budget transfer from national to province.

“*Once the HOD* [Head of Department] *has signed, you would expect implementation to be smooth because this is an approved business plan. However, implementing and spending remained a challenge. Whatever internal processes of the province were in place at the time, it also, affected us. If the province had problems paying suppliers, it included our* [ICRM] *budget*.” *(Key informant 5, District level, GP)*

The insufficient resources affected the morale of policy actors and constrained ICRM programme implementation.

### Specific context

Three major themes emerged, namely gaps in the existing policy (NCS), insufficient policy coherence or disjuncture between the NCS and ICRM programme, and a need to consider geographical variations when implementing a national policy. The majority of key informants were of the opinion that the ICRM programme is more appropriate for the PHC level, compared to the NCS, but that the ICRM programme could assist with compliance with the NCS.

“*The national core standards* [NCS] *were established initially from a hospital perspective and didn't necessarily address quality issues related to a PHC facility. Therefore, it* [ICRM] *helped to refine the tools for the OHSC in terms of how they measure quality in a* [primary] *health care setting.*” (Key informant 5, District, GP)

Some key informants were of the opinion that there was insufficient communication on and lack of enforcement of the NCS, both of which could have achieved the goals of improving PHC service quality.

“*If we emphasised the concept of national core standards, facilities would actually be ideal. But I think implementation was lacking.*” (Key informant 7, Tshwane Municipality, GP)

Although some key informants viewed the ICRM programme as more appropriate to address quality standards at PHC facilities, they pointed to several design problems. These included duplication rather than synergies between the ICRM programme and the NCS, competing processes, different expectations, different indicators, tools and measurement approaches, at times requiring different kinds of evidence.

“*There are still some challenges and clashes between the two* [NCS and ICRM programme]*, because you find that there are some disagreements in relation to what is required from a facility to comply with both NCS and ICRM programme. They* [NCS and ICRM Programme Tools] *want different evidence in relation to the same element.*” (Key informant 2, Provincial level, MP)

“*There are times where you find that the NCS would say this is not necessary for the primary healthcare setup. And then the ICRM programme says this is an important or an essential or a vital element. The systems are conflicting with each other in terms of scoring.*” (Key informant 12, Sub-district, GP)

The district and local government actors reported that the duplication of efforts to comply with the ICRM programme and the NCS led to frustration, confusion, “*audit exhaustion*” and demotivation, exacerbated by resource constraints.

“*Some implications as I’m saying it’s audit exhaustion*” (Key informant 7, Tshwane Municipality, GP)

Key informants were of the opinion that that they could not influence the alignment between the NCS and ICRM programme tools because they did not have the power to do so.

“*We have raised these issues* [of merging NCS and ICRM programme tools] *in different platforms, and it is not helping us at all.*” (Key informant 5, District level, GP)

“*The debate to align the tools took full two years, I’m not exaggerating*.” (Key informant 2, National)

Key informants also highlighted differences between rural and urban facilities, and a need to consider such geographical variations during implementation. They pointed to the facilities that were not designed for expanded PHC services, yet were expected to meet the desired programme standards.

“*A national tool can’t be a one size fits all process. While it can set generic principles, there are provincial issues that need to be dealt with, and for example the level of services, staffing and skills that you will have in a clinic in a rural setting would be different from an urban setting.*” (Key Informant 5, District level, GP)

“*We have facilities that were not custom made to be clinics, and we are trying to force a tool to evaluate them as ideal when they are actually not.*” (Key Informant 24, District level, MP)

Some expressed a sense of despair on whether it was possible to implement the ICRM programme in PHC clinics with infrastructure challenges.

“*I have three facilities that will never be ideal* [meet the requirements of the ICRM programme]*. It’s one facility in a one room house; it’s another clinic that is situated in an old hotel that is dilapidated, and* [the third] *facility that is situated in a farming area.*” (Key Informant 22, District, MP)

## Discussion

This study set out to generate new knowledge on the policy context of the ICRM programme implementation and examine the influence of context on the characteristics of the policy actors and how these interactions influenced ICRM implementation. Furthermore, we explored the policy coherence between the ICRM programme and the NCS as well as the perceptions of national, provincial and local government stakeholders on these two quality improvement reforms.

This was one of the first studies in South Africa that used Bressers’ Contextual Interaction Theory to explore the implementation of the ICRM programme. The latter is a relatively recent reform, implemented since 2013. This study found that the NDoH’s primary motivation (the wider context) was PHC quality improvement in preparation for the implementation of the NHI system. PHC is the stated foundation of the South African health system [51]. Notwithstanding the existence of the OHSC as a legal entity, the NDoH used its legal authority and power to conceptualise the ICRM programme as the main vehicle for PHC quality improvement and to ensure compliance with the NCS [8].

In this study, the structural context of the ICRM programme implementation was characterised by contestations about the roles and responsibilities of the different government spheres. These contestations were illustrated by conflicting views of key informants and perceptions of unfulfilled responsibilities or commitments, lack of enforcement of existing policies, such as the NCS, and unfunded mandates. These contestations were exacerbated by reportedly weak intergovernmental relationships and poor or ineffective communication. However, the South African Constitution is clear on the roles and responsibilities of different government spheres [6], while sectoral legislation, such as the National Health Act [7] and Intergovernmental Relations Framework Act [52], provides additional clarity. The ICRM manual also outlines the roles and responsibilities of different government spheres in the implementation of the programme [12].

Other studies that have examined the effectiveness of local government in South Africa have also found that insufficient clarity on the roles and responsibilities across government spheres led to overlapping functions by provincial and national government, with adverse consequences for social service delivery at the local level, complaints of unfunded mandates and lack of accountability to communities [53, 54]. Health policy implementation studies in South Africa have underscored the importance of role clarity of different government spheres in PHC delivery [55]. In contrast, poor communication [36, 37] and weak or strained relationships across different government spheres hamper implementation and the effective functioning of the district health system [55–58]. These findings on the lack of role clarity and diffuse accountability have also been reported elsewhere. A study in Nigeria also found overlapping functions and diffuse accountability when many ministries were assigned an administrative role at the PHC level [59], while a multi-country study in Mozambique, Nepal and Rwanda underscored the importance of effective governance in ensuring successful implementation of maternal and child health policies [60].

Our study found that local leadership enabled ICRM implementation as the experienced and knowledgeable managers were able to motivate or negotiate for additional resources to accelerate implementation. Other studies in South Africa [61], Bangladesh, Ethiopia, Kenya and India [62] have highlighted the importance of leadership for successful policy implementation. A study in Ghana also found that managers’ capacity to lead is directly influenced by the contextual factors [63]. In Indonesia, a study on factors influencing the success of a PHC quality improvement programme found that strong local leadership and their involvement in resource decision-making were success factors [64].

In our study, some key informants complained about insufficient resources despite the allocation of a conditional grant to the NHI pilot districts; they also had concerns about long-term sustainability of the ICRM programme. These key informants’ perceptions could be linked to the insufficient or unclear communication about the programme or their resentment about the reported lack of consultation and involvement of policy actors at provincial and local government. Studies in other countries have also found that resource constraints adversely affect policy implementation. In Nigeria, two studies found that insufficient funding, incompetence in financial management, a weak drug supply system, inadequate infrastructure and weak coordination of resources hampered the implementation of PHC policies [65, 66]. Similarly, in Tanzania, the dependency on a centralised budget for accessing funds delayed the implementation of the contracting-out of PHC services [67] while, in Ghana, inflexible funds led to a disjuncture between the planned budget and actual expenditure [68].

Key informants in our study reported that the NDoH did not consider geographical variations and/or the gaps in the NCS. A study on management of health equity in Ethiopia found that geographical variations influenced inequalities in access to healthcare and the degree of support and supervision of frontline facility managers [69].

Bressers’ Contextual Interaction Theory highlights the dynamic relationship between the context and the motivation, cognition and power of policy actors [70]. The theory underscores the importance of resources because resource availability could strengthen whereas shortages could weaken the power and motivation of a given policy actor [70]. In this study, the resource constraints (whether real or perceived) appeared to have weakened the position of policy actors at provincial, district and local government. Although these policy actors engaged in programme implementation, they expressed a mix of feelings of frustration, exhaustion, demotivation, resentment (because of lack of involvement) and disempowerment and they could influence the sustainability of the ICRM programme. For example, the 2017 ICRM programme national report indicated that 16% of facilities that had previously attained ideal clinic status had lost it [16]. The 2019 annual report from the NDoH indicated that 44% of the PHC facilities in South Africa had not met the ICRM programme requirements since its inception [17]. On February 2020, a 5-year review of the ICRM programme indicated that a total of 623 (25%) of the facilities that had attained the ideal clinic status since the programme started had lost their status [18].

We also aimed to explore whether there is policy coherence between the ICRM programme and the NCS of the OHSC as both these reforms focus on health system-wide quality improvements. Linked to this, how stakeholders at national, provincial and local government view or interpret these two quality improvement reforms was explored. The study found that there was insufficient policy coherence between the ICRM programme and the NCS of the OHSC. Although the ICRM programme was conceptualised to deal with the inability of the PHC facilities to comply with the NCS, the ICRM programme appeared to have developed in parallel fashion, with little interaction or synergies between the two quality of care reforms. The policy disjuncture between the ICRM programme and the national core quality standards led to a duplication of efforts, conflicting standards and tools, confusion, and exhaustion among the front-line staff responsible for implementation. Some key informants reported that neither provincial/local government nor front-line staff had the power to influence the alignment of ICRM tools, contributing to feelings of despair and demotivation. The study found that stakeholders at national, provincial and local government were in agreement on their views about a lack of coherence of the NCS and the ICRM a programme, yet they verbalised having no power to influence the alignment.

Scholars have highlighted the confusion created among implementers when there is a lack of policy coherence or policy disjuncture, with possible duplicated efforts and wastage of scarce resources [14, 71, 72]. Hewlett suggested that alignment of a new policy with an existing policy on a similar issue should be considered during policy design [73]. Another South African study found policy incoherence between government action to promote a healthy food supply and the government objectives for economic liberalisation [74]. A study in Iran found that power dynamics, disagreements and diverse interests among policy-makers and organisations resulted in policy incoherence [75]. Similarly, a multi-country study in China, India and Vietnam also found a lack of coherence between each country’s human resource strategy and maternal and child health policy, which adversely affected implementation [76].

The study was limited by focusing on two of the nine South African provinces. The findings may not reflect the implementation of the ICRM programme in the other provinces. In addition, key informants were interviewed at a point in time that may have influenced the views expressed. There is always the possibility of social desirability bias because of the self-reported nature of the information. However, the PR ensured that care was taken with the design of the interview schedule. The interview was conducted in a private and comfortable setting and rapport was established with each key informant. Importantly, all the key informants are senior government officials, whereas the PR is a relatively junior doctoral student.

However, the study has numerous strengths; this was one of the first studies in South Africa that applied Bressers’ Contextual Interaction Theory to the implementation of the ICRM programme in the health sector. We generated new knowledge on the policy context of the ICRM programme implementation and the influence of this context on actors’ motivation, cognition and power. The study yielded rich information on the complexities and nuances of ICRM policy implementation in these two NHI pilot districts and the unique issues that need to be addressed to strengthen implementation.

There are several recommendations that emerge from this study. With regard to the policy disjuncture, the South African government has invested considerable resources into both the ICRM programme and the OHSC as a legal entity. The ICRM programme has the potential to revolutionise quality at the PHC level and to be complementary to the NCS of the OHSC. Careful planning, coordination, proactive leadership, resources and skills are policy implementation enablers [72, 73]. The policy incoherence could be addressed by improving communication across the three spheres of government. The South African Lancet National Commission report provides a useful starting point for the discussion across national, provincial and local government on the critical imperative of improving quality in the South African health system [38]. The policy incoherence could also be addressed by involving frontline PHC and district managers and incorporating their views in the revision and alignment of the tools used in the ICRM programme and the NCS. At the time of writing, there were encouraging developments with staff responsible for both programmes having discussions about the alignment of the assessment criteria and tools.

The NDoH has the opportunity to improve leadership of the ICRM. We recommend that the NDoH should develop a clear communication strategy, including an outline of different roles and responsibilities. The NDoH should also review the criteria for any future conditional grants to ensure that the guidelines are clear, that the views of all policy actors are considered and that flexibility is built into the grant.

Lastly, the NDoH should invest in broader capacity-building to ensure successful implementation of the ICRM programme. Such capacity-building should be at the health system level through the creation of an enabling environment with infrastructure and resources and at an individual level through the training of district and frontline PHC managers.

Future research is needed on the impact of the ICRM programme implementation, using quantitative indicators and covering all provinces to ensure generalisability. The qualitative information generated in this study could assist with the design of a questionnaire to measure impact.

## Conclusion

This study generated new knowledge on the context of implementing the ICRM programme, a health sector reform that involves PHC quality improvements. South Africa is preparing for the full implementation of the NHI system. The study contributes lessons for policy-makers to consider the context of policy implementation and how the context influences the characteristics of policy actors. The design of any health reform should consider similar policies or initiatives to ensure coherence and the availability of resources. Any major change initiative such as the ICRM programme requires involvement of all relevant policy actors in both design and implementation. A clear communication strategy and ongoing monitoring and evaluation are prerequisites for the success of policy implementation.

## Data Availability

This was a qualitative study, all data generated from the participants interviews has been cited verbatim within the manuscript in the results section.

## Abbreviations

GDoH: Gauteng Department of Health
GP: Gauteng Province
ICRM: Ideal Clinic Realisation and Maintenance
LMIC: low- and middle-income countries
MP: Mpumalanga Province
NCS: National Core Standards
NDoH: National Department of Health
NHI: National Health Insurance
OHSC: Office of Health Standards Compliance
PHC: primary healthcare
PR: principal researcher
UHC: Universal health coverage

## References

[1] United Nations. Transforming Our World: The 2030 Agenda for Sustainable Development. New York: United Nations; 2015.

[2] World Health Organization (2018). Draft Thirteenth General Programme of Work, 2019–2023. Report by the Director-General. World Health Assembly Seventy-first World Health Assembly, 71/4, Provisional agenda item 11.1.

[3] World Health Organization (2018). Organisation for Economic Co-operation and Development, World Bank. Delivering Quality Health Services: A Global Imperative for Universal Health Coverage.

[4] National Academies of Sciences Engineering and Medicine (2018). Crossing the Global Quality Chasm: Improving Health Care Worldwide.

[5] Kruk ME, Gage AD, Arsenault C, Jordan K, Leslie HH, Roder-DeWan S, et al. High-quality health systems in the Sustainable Development Goals era: time for a revolution. Lancet Glob Health. 2018;6:E1196–252. 10.1016/S2214-109X(18)30386-3. 10.1016/S2214-109X(18)30386-3 PMC7734391 30196093

[6] Republic of South Africa (1996). The Constitution of the Republic of South Africa.

[7] Republic of South Africa (2003). National Health Act no. 61 of 2003.

[8] National Department of Health (2017). National Health Insurance for South Africa: Towards Universal Health Coverage.

[9] Republic of South Africa (2013). National Health Amendment Act no. 12 of 2013.

[10] Steinhobel R, Massyn N, Peer N (2015). The Ideal Clinic Programme 2015/16.

[11] National Department of Health (2016). Ideal Clinic Manual version 16.

[12] National Department of Health (2017). Ideal Clinic Manual version 17.

[13] Moat KA, Lavis JN, Abelson J (2013). How contexts and issues influence the use of policy-relevant research syntheses: a critical interpretive synthesis. Milbank Q.

[14] May PJ, Sapotichne J, Workman S. Policy coherence and policy domains. Policy Stud J. 2006;34(3):381–403. 10.1111/j.1541-0072.2006.00178.x.

[15] Vinke-de Kruijf J, Teodosiu C, Bressers H, De Boer C, Vinke-de Kruijf J, Özero G, Bressers HTA (2013). How contextual factors influence the effectiveness of international projects: The case of Dutch-funded fl ood risk management projects in Romania. Water Governance, Policy and Knowledge Transfer.

[16] Steinhobel R, Massyn N, Padarath A, Peer N, Day C (2017). PHC Management. District health barometer 2017/2018.

[17] National Department of Health (2019). National Department of Health Annual Report.

[18] National Department of Health. A National Report of ICRM Programme 5-year Review. The National 5 Year Ideal Clinic Realisation and Maintenance Programme Review and Quality Assurance Workshop. Johannesburg: NDoH; 2020. https://www.idealhealthfacility.org.za/. Accessed 25 May 2020.

[19] Howlett M. The lessons of failure: learning and blame avoidance in public policy-making. Int Polit Sci Rev. 2012;33(5):539–55. 10.1177/0192512112453603.

[20] Howlett M, Ramesh M, Wu X (2015). Understanding the persistence of policy failures: the role of politics, governance and uncertainty. Public Policy Admin.

[21] Walt G, Shiffman J, Schneider H, Murray SF, Brugha R, Gilson L (2008). ‘Doing’ health policy analysis: Methodological and conceptual reflections and challenges. Health Policy Plann.

[22] Rao KD, Arora R, Ghaffar A (2014). Health systems research in the time of health system reform in India: a review. Health Res Policy Syst.

[23] Collins T (2005). Health policy analysis: a simple tool for policy makers. Public Health.

[24] Collins C, Green A, Hunter D (1999). Health sector reform and the interpretation of policy context. Health Policy.

[25] Mohlakoana N (2014). Implementing the South African Free Basic Alternative Energy Policy: A Dynamic Actor Interaction. PhD thesis.

[26] Okungu V, Chuma J, McIntyre D (2017). The cost of free health care for all Kenyans: Assessing the financial sustainability of contributory and non-contributory financing mechanisms. Int J Equity Health.

[27] Koduah A, van Dijk H, Agyepong IA (2015). The role of policy actors and contextual factors in policy agenda setting and formulation: maternal fee exemption policies in Ghana over four and a half decades. Health Res Policy Syst.

[28] Gilson L, Doherty J, Lake S, McIntyre D, Mwikisa C, Thomas S (2003). The SAZA study: implementing health financing reform in South Africa and Zambia. Health Policy Plann..

[29] Ramani S, Sivakami M, Gilson L (2019). How context affects implementation of the Primary Health Care approach: an analysis of what happened to primary health centres in India. BMJ Glob Health.

[30] Djellouli N, Quevedo-Gómez MC (2015). Challenges to successful implementation of HIV and AIDS-related health policies in Cartagena, Colombia. Soc Sci Med.

[31] McCord R, Cronk R, Tomaro J, Reuland F, Behnke N, Mmodzi Tseka J (2019). The implementation of environmental health policies in health care facilities: the case of Malawi. Int J Hyg Environ Health.

[32] Moyo L, Wehn U. Interaction dynamics: the case of the water sector skills plan in South Africa. Eval Program Plann. 2017;60:91–9. 10.1016/j.evalprogplan.2016.08.021. 10.1016/j.evalprogplan.2016.08.021 27792898

[33] Ditlopo P, Blaauw D, Rispel L, Thomas S, Bidwell P (2013). Policy implementation and financial incentives for nurses in two South African provinces: a case study on the occupation-specific dispensation. Glob Health Action.

[34] Ditlopo P, Blaauw D, Bidwell P, Thomas S (2011). Analyzing the implementation of the rural allowance in hospitals in North West Province, South Africa. J Health Policy.

[35] Rispel LC. Transforming nursing policy, practice and management in South Africa, Glob Health Action. 2015;8. 10.3402/gha.v8.28005. 10.3402/gha.v8.28005 PMC4430687 25971403

[36] Blaauw D, Ditlopo P, Rispel L. Nursing education reform in South Africa – lessons from a policy analysis study. Glob Health Action. 2014;7(26401). 10.3402/gha.v7.26401. 10.3402/gha.v7.26401 PMC4275647 25537941

[37] Blaauw D, Penn-Kekana L, Rispel L. Contestations and complexities of nurses participation in policymaking in South Africa. Glob Health Action. 2014;7(25327). 10.3402/gha.v7.25327. 10.3402/gha.v7.25327 PMC4275627 25537938

[38] South African Lancet National Commission (2019). Confronting the Right to Ethical and Accountable Quality Health Care in South Africa: A Consensus Report.

[39] National Department of Health (2017). National Health Insurance Policy: Towards Universal Health Coverage.

[40] Hunter JR, Chandran TM, Asmall S, Tucker J-M, Ravhengani NM, Mokgalagadi Y (2017). The Ideal Clinic in South Africa: progress and challenges in implementation. South Afr Health Rev.

[41] Bressers H, Nahrath S, Varone F (2009). From public administration to policy networks: contextual interaction analysis. Rediscovering Public Law and Public Administration in Comparative Policy Analysis: A Tribute to Peter Knoepfel.

[42] Bressers H, Bressers N, Kuks S, Larrue C, Bressers H, Bressers N, Larrue C (2016). The governance assessment tool and its use. Governance for Drought Resilience.

[43] Bressers H, Bressers N, Larrue C (2016). Governance for drought resilience. The Governance Assessment Tool and Its Use.

[44] National Department of Health (2015). National Health Insurance in South Africa: Towards Universal Health Coverage Version 40.

[45] Statistics South Africa (2018). Provincial Profile: Community Survey.

[46] Gauteng Department of Health (2018). Tshwane District Health Plan 2018/2019–2020/2021.

[47] City of Tshwane Municipality. Health Services. http://www.tshwane.gov.za/sites/Departments/Health-Department/Pages/Health-Service.aspx. Accessed 1 Apr 2020 .

[48] Mpumalanga Department of Health. Mpumalanga Department of Health – Annual Perfomance Report. 2017. http://www.mpuhealth.gov.za/AnnualReport/Dept%20of%20Health%20Annual%20Report%202017.pdf. Accessed 21 Apr 2020.

[49] Nowell LS, Norris JM, White DE, Moules NJ (2017). Thematic analysis: striving to meet the trustworthiness criteria. Int J Qual Methods.

[50] Health Systems Trust (2012). The National Health Care Facilities Baseline Audit: National Summary Report.

[51] Rispel LC, Moorman J, Munyewende P, Meyiwa T, Nkondo M, Chitiga-Mabugu M, Sithole M, Nyamnjoh F (2014). Primary health care as the foundation of the South African health system: Myth or reality?. State of the Nation South Africa 2014. South Africa 1994–2014: A twenty-year review.

[52] Republic of South Africa (2005). Intergovernmental Relations Framework Act no. 13 of 2005.

[53] Steytler N, Fessha YT (2007). Defining local government powers and functions. S Afr Law J.

[54] Christmas A, De Visser J (2009). Bridging the gap between theory and practice: reviewing the functions and powers of local government in South Africa. Commonw J Local Gov.

[55] Rispel L (2016). Analysing the progress and fault lines of health sector transformation in South Africa. South African Health Review.

[56] Ile IU (2010). Strengthening intergovernmental relations for improved service delivery in South Africa: Issues for consideration. J US-China Public Admin.

[57] Brooke-Sumner C, Petersen-Williams P, Kruger J, Mahomed H, Myers B. ‘Doing more with less’: a qualitative investigation of perceptions of South African health service managers on implementation of health innovations. Health Policy Plann. 2019;34(2):132–40. 10.1093/heapol/czz017. 10.1093/heapol/czz017 PMC6481285 30863845

[58] Kredo T, Abrams A, Young T, Louw Q, Volmink J, Daniels K (2017). Primary care clinical practice guidelines in South Africa: qualitative study exploring perspectives of national stakeholders. BMC Health Serv Res.

[59] George AS, Erchick DJ, Zubairu MM, Barau IY, Wonodi C (2016). Sparking, supporting and steering change: grounding an accountability framework with viewpoints from Nigerian routine immunization and primary health care government officials. Health Policy Plan.

[60] Samuels F, Amaya AB, Balabanova D (2017). Drivers of health system strengthening: Learning from implementation of maternal and child health programmes in Mozambique, Nepal and Rwanda. Health Policy Plan.

[61] Schneider H, Nxumalo N (2017). Leadership and governance of community health worker programmes at scale: a cross case analysis of provincial implementation in South Africa. Int J Equity Health.

[62] Nisbett N, Wach E, Haddad L, El Arifeen S (2015). What drives and constraints effective leadership in tackling child undernutrition? Findings from Bangladesh, Ethiopia, India and Kenya. Food Policy.

[63] Aberese-Ako M, Agyepong IA, van Dijk H (2018). Leadership styles in two Ghanaian hospitals in a challenging environment. Health Policy Plann.

[64] Limato R, Tumbelaka P, Ahmed R, Nasir S, Syafruddin D, Ormel H (2019). What factors do make quality improvement work in primary health care? Experiences of maternal health quality improvement teams in three Puskesmas in Indonesia. PLoS One.

[65] Ogbuabor DC, Onwujekwe OE (2018). Implementation of free maternal and child healthcare policies: assessment of influence of context and institutional capacity of health facilities in south-east Nigeria. Glob Health Action.

[66] Tilley-Gyado R, Filani O, Morhason-Bello I, Adewole IF (2016). Strengthening the primary care delivery system: A catalytic investment toward achieving universal health coverage in Nigeria. Health Syst Reform.

[67] Maluka S, Chitama D, Dungumaro E, Masawe C, Rao K, Shroff Z (2018). Contracting-out primary health care services in Tanzania towards UHC: how policy processes and context influence policy design and implementation. Int J Equity Health.

[68] Mutale W, Chintu N, Amoroso C, Awoonor-Williams K, Phillips J, Baynes C (2013). Improving health information systems for decision making across five sub-Saharan African countries: implementation strategies from the African Health initiative. BMC Health Serv Res.

[69] Bergen N, Ruckert A, Kulkarni MA, Abebe L, Morankar S, Labonté R (2019). Subnational health management and the advancement of health equity: a case study of Ethiopia. Glob Health Res Policy.

[70] Owens KA, Bressers H (2013). A comparative analysis of how actors implement: Testing the contextual interaction theory in 48 cases of wetland restoration. J Compar Policy Anal Res Pract.

[71] Cejudo GM, Michel CL (2017). Addressing fragmented government action: Coordination, coherence, and integration. Policy Sci.

[72] Kern F, Kivimaa P, Martiskainen M (2017). Policy packaging or policy patching? The development of complex energy efficiency policy mixes. Energy Res Soc Sci.

[73] Howlett M, Rayner J (2013). Patching vs packaging in policy formulation: assessing policy portfolio design. Politics Governance.

[74] Thow A-M, Greenberg S, Hara M, Friel S, Sanders D (2018). Improving policy coherence for food security and nutrition in South Africa: a qualitative policy analysis. Food Security.

[75] Danaeefard H, Ahmadi H, Pourezzat AA (2019). Expert consensus on factors reducing policy coherence in the context of Iran: Delphi-AHP. Int J Public Adm.

[76] Martineau T, Mirzoev T, Pearson S, Ha BTT, Xu Q, Ramani KV (2013). Coherence between health policy and human resource strategy: lessons from maternal health in Vietnam, India and China. Health Policy Plan.

